# Streptococcus Intermedius Brain and Diverticular Abscesses After Dental Manipulation: A Case Report

**DOI:** 10.7759/cureus.2061

**Published:** 2018-01-13

**Authors:** Hassan Al Moussawi, Michael Krzyzak, Zainab Awada, Jean M Chalhoub

**Affiliations:** 1 Department of Medicine, Staten Island University Hospital, Northwell Health

**Keywords:** brain abscess, streptococcus intermedius, diverticular abscess

## Abstract

A brain abscess is defined as a focal intracerebral infection consisting of an encapsulated collection of pus, which can be a life-threatening complication of infections, trauma, or surgery. While immunocompromised patients can have a wide array of causative organisms, bacterial species represent the most common etiology in immunocompetent individuals. The incidence of brain abscesses ranges from 0.4 to 0.9 per 100,000, with a high predisposition among immunocompromised patients and in those with disruption of the blood-brain barrier.

The most common causative organisms found were Streptococcus species, particularly S. viridians and S. pneumonia, Enterococcus, and Staphylococcus species, mainly S. aurieus and S. epidermidis. Microorganism can invade the brain through different mechanisms, either directly by contiguous spread and odontogenic infections, which usually cause a single brain abscess, or indirectly through hematogenous spread which can cause multiple brain abscesses. Both surgical and conservative dental procedures contribute to hematogenous spreading of oral microorganisms. Although most of those organisms are eliminated shortly after they gain access to the bloodstream, some can persist and contribute to the pathogenesis of abscesses in the appropriate environment. Odontogenic origins are rarely implicated in the formation of brain abscesses, and oral foci comprise approximately 5% of identified cases. We report a case of brain and diverticular abscesses due to S. intermidius occurring two months after dental extraction. This case highlights the fact that even usual dental workup can result in the development of bacteremia and disseminated abscesses including but not restricted to the brain. Consequently, in addition to identifying the possible source of bacteremia with an extensive history and physical exam, the diagnosis of Streptococcus milleri organisms should prompt the physicians to screen for sites of possible metastatic infection spread.

## Introduction

Streptococcus intermedius (S. intermedius) is a gram-positive oral bacterium that is part of the Streptococcus milleri group. It is a known inhabitant of the oral cavity as well as the upper-respiratory, gastrointestinal, and female urogenital tracts [1]. Although this organism is considered part of the normal flora, it can also present as an opportunistic pathogen. It is a common cause of brain abscesses, liver abscesses, and other serious purulent infections. Multiple comorbidities, malignancy, and diabetes are widely known to increase the risk of infection with Streptococcus intermedius [2].

Cases of S. intermedius brain abscesses reported previously were associated with an active oral infection. To our knowledge, this is the first case of concomitant brain and diverticular abscess to be reported two months after routine dental work.

## Case presentation

A 56-year-old woman with a history of ductal carcinoma in-situ status post lumpectomy and radiation therapy in 2006, hypothyroidism, and diverticulosis, presented for dizziness and headaches for two days. Her headaches had been intermittently present for a few weeks but were progressively worsening, up until the week of admission when she began having blurry vision, dizziness, and what she reported as the worst headache of her life. There was no history of falls or trauma, and no constitutional symptoms were reported. The patient’s review of systems was otherwise negative except for a tooth extraction two months prior to presentation. Still, the patient had no oral or pharyngeal complaints, no signs of oral wound infection, no pus or erythema, and no tenderness.

**Figure 1:**
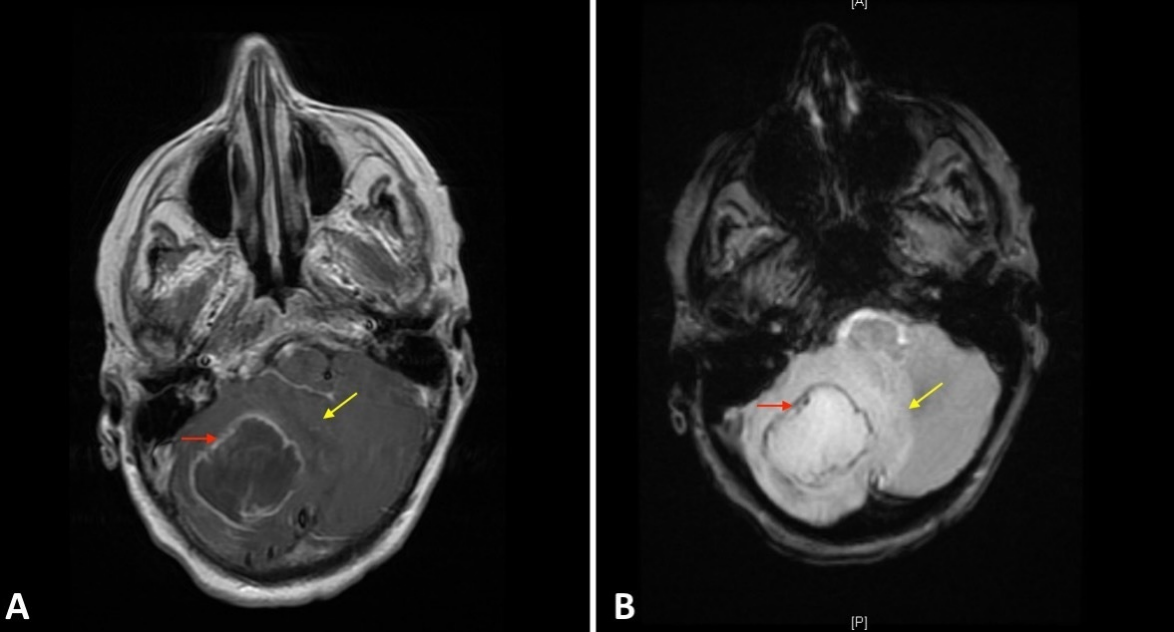
Imaging Findings A 4.6 cm abscess with rim-enhancing lesion in the right cerebellar hemisphere (red arrows) and extensive surrounding edema (yellow arrows) seen on (A) T1 post contrast and (B) diffusion weighted imaging (DWI).

On admission to the hospital, a computed tomography (CT) scan of the head showed a 3.9 x 2.7 x 2.3 cm hypodense right cerebellar mass with adjacent edema. Given a history of breast cancer, findings were interpreted to represent metastatic disease. The associated mass effect was noted on the fourth ventricle with resultant obstructive hydrocephalus. The patient was admitted to the intensive care unit (ICU) for neurosurgical evaluation. Magnetic resonance imaging (MRI) of the brain one day after presentation showed a 4.6 cm rim-enhancing lesion with extensive surrounding edema. Imaging features (i.e. central restricted diffusion) were in favor of an abscess (Figure 1). Within the first 48 hours, temperatures up to 102°F were reported, and as a result, vancomycin, ceftriaxone, and metronidazole were started empirically. Two days after admission, the patient was taken to the operating room, and 22 milliliters of pus were drained, growing Streptococcus intermedius on culture. Due to persistent fevers and a lack of clinical improvement, the patient underwent another surgery for drainage of the remaining abscess contents.

**Figure 2:**
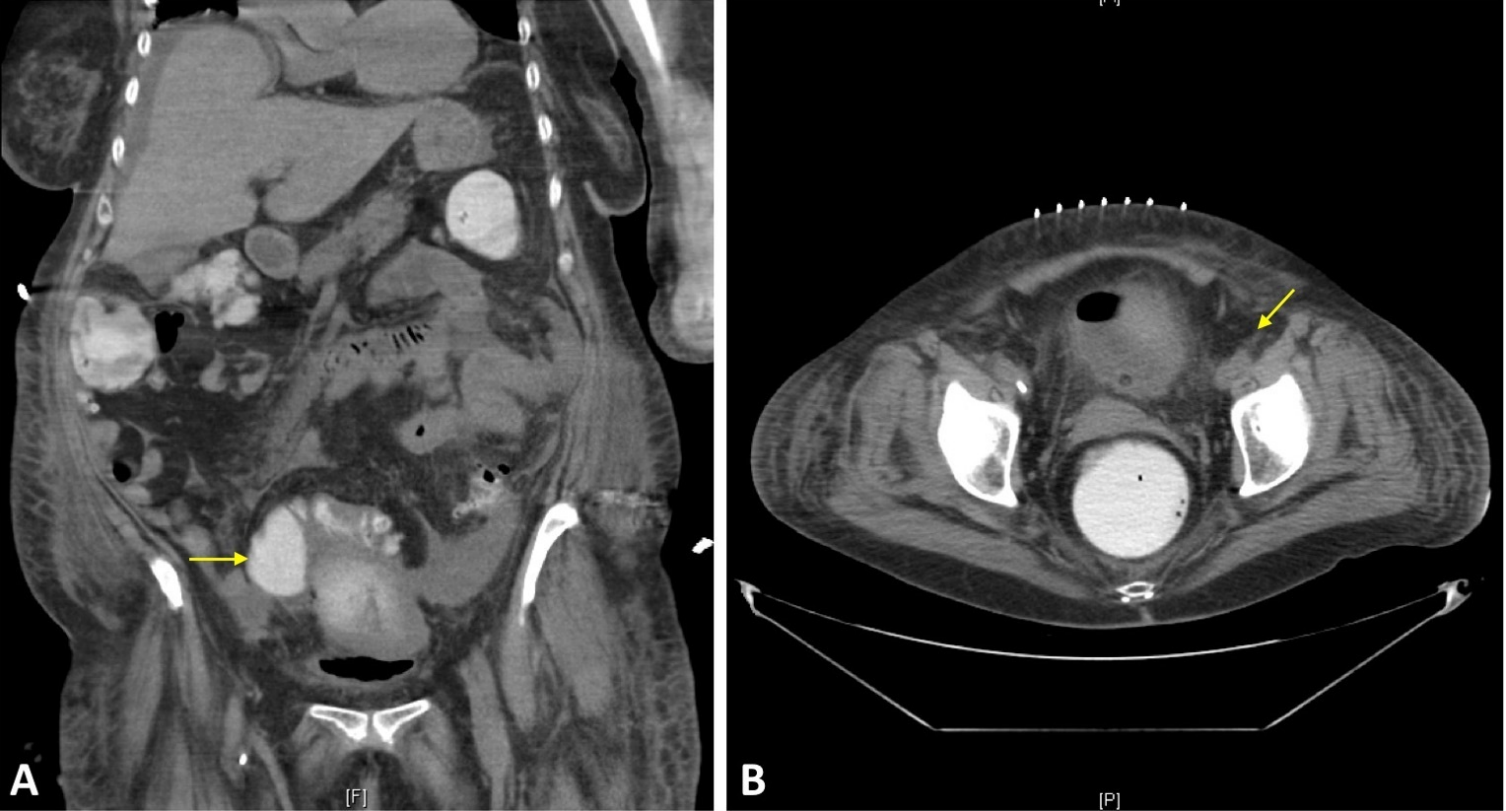
Diverticular Abscess on Computed Tomography (CT) Imaging CT scan showing sigmoid diverticulitis with surrounding inflammation and an adjacent lobulated 6.9 x 5.4 x 6.1 cm abscess (yellow arrow) on (A) coronal and (B) axial sections.

However, the patient continued to be febrile, and additional foci were sought. An oropharyngeal examination by the dental team yielded no nidus of infection. An abdominal CT scan found sigmoid diverticulitis with surrounding inflammation as well as an adjacent lobulated 6.9 x 5.4 x 6.1 cm abscess (Figure 2). Surgical drainage was performed, and cultures also showed Streptococcus intermedius. The patient had a complete recovery thereafter.

## Discussion

Brain lesions are either infectious, inflammatory, neoplastic, or vascular. Given the patient’s history of breast cancer in situ, metastatic cancer was highly considered in the differential diagnosis. With the increasing rate of the five-year survival rate in breast cancer from 75% to 91% in 2010 [3], the chance of having recurrences or metastasis becomes higher. On the other hand, brain abscesses have decreased on the clinical differential with the advent of antimicrobials [4].

Nonetheless, Streptococcus intermedius is still a relatively common cause of brain abscesses. It is known to cause abscesses in the head, neck, central nervous system, respiratory tract, gastrointestinal tract, genitourinary tract, skin, and soft tissues. S. intermedius produces multiple hydrolytic enzymes such as galactosidase, P-D-fucosidase, PN-acetylglucosaminidase, N-acetylgalactosaminidase, a t-D-glucosidase, sialidase, and hyaluronidase, which help the bacteria in destroying the extracellular matrix of the connective tissues. Moreover, the presence of surface protein antigen I/II help the pathogen in adhering to fibronectin and laminin [5]. Those two characteristics are believed to be the most important virulence factors associated with the increased pathogenicity of S. intermedius, manifested in the formation of deep abscesses.

Due to the recent dental work, it can be postulated that bacterial dislocation to the blood occurred, leading to a hematogenous spread into the brain and abdomen. Fever and headaches comprise only two of the three presenting symptoms of the abscess triad, which also includes focal neurologic defects. Diagnosis is often challenging and requires a high index of suspicion. Diffusion-weighted (DWI) MRI can be helpful and is capable of differentiating ring-enhancing lesions due to brain abscess from neoplastic lesions.

S. intermedius is susceptible to several antibiotic classes including cephalosporins, penicillins, and lincosamides. Antibiotics, abscess drainage, and surgical excision are the mainstay of treatment. Surgical therapy is considered in multi-loculated, fungal, traumatic abscesses, or in the case that no treatment response was recorded within a week of treatment initiation. Additionally, complete surgical drainage is recommended for abscesses greater than 2.5 cm. Eventually, six to eight weeks of antibiotics are recommended based upon the causative organism and its resistance patterns [6]. Appropriate timely identification and treatment of brain abscesses are crucial as this reduces mortality from 40% to 10% [7].

## Conclusions

In conclusion, brain abscesses should be considered in cases of fever, headache, and neurologic defects after recent dental workup. A careful history and physical exam should be performed to identify the etiology of the causative agent and to screen for potential involvement of other organs.
